# Metabolomics Unravel Contrasting Effects of Biodiversity on the Performance of Individual Plant Species

**DOI:** 10.1371/journal.pone.0012569

**Published:** 2010-09-07

**Authors:** Christian Scherling, Christiane Roscher, Patrick Giavalisco, Ernst-Detlef Schulze, Wolfram Weckwerth

**Affiliations:** 1 Department of Bioinformatics and Biochemistry, Technical University of Braunschweig, Braunschweig, Germany; 2 Max Planck Institute for Biogeochemistry, Jena, Germany; 3 Department of Community Ecology, Helmholtz Centre for Environmental Research, Halle, Germany; 4 Max Planck Institute of Molecular Plant Physiology, Potsdam, Germany; 5 Department of Molecular Systems Biology (MOSYS), University of Vienna, Vienna, Austria; Centre National de la Recherche Scientifique, France

## Abstract

In spite of evidence for positive diversity-productivity relationships increasing plant diversity has highly variable effects on the performance of individual plant species, but the mechanisms behind these differential responses are far from being understood. To gain deeper insights into the physiological responses of individual plant species to increasing plant diversity we performed systematic untargeted metabolite profiling on a number of herbs derived from a grassland biodiversity experiment (Jena Experiment). The Jena Experiment comprises plots of varying species number (1, 2, 4, 8, 16 and 60) and number and composition of functional groups (1 to 4; grasses, legumes, tall herbs, small herbs). In this study the metabolomes of two tall-growing herbs (legume: *Medicago x varia*; non-legume: *Knautia arvensis*) and three small-growing herbs (legume: *Lotus corniculatus*; non-legumes: *Bellis perennis*, *Leontodon autumnalis*) in plant communities of increasing diversity were analyzed. For metabolite profiling we combined gas chromatography coupled to time-of-flight mass spectrometry (GC-TOF-MS) and UPLC coupled to FT-ICR-MS (LC-FT-MS) analyses from the same sample. This resulted in several thousands of detected m/z-features. ANOVA and multivariate statistical analysis revealed 139 significantly changed metabolites (30 by GC-TOF-MS and 109 by LC-FT-MS). The small-statured plants *L. autumnalis*, *B. perennis* and *L. corniculatus* showed metabolic response signatures to increasing plant diversity and species richness in contrast to tall-statured plants. Key-metabolites indicated C- and N-limitation for the non-leguminous small-statured species *B. perennis* and *L. autumnalis*, while the metabolic signature of the small-statured legume *L. corniculatus* indicated facilitation by other legumes. Thus, metabolomic analysis provided evidence for negative effects of resource competition on the investigated small-statured herbs that might mechanistically explain their decreasing performance with increasing plant diversity. In contrast, taller species often becoming dominant in mixed plant communities did not show modified metabolite profiles in response to altered resource availability with increasing plant diversity. Taken together, our study demonstrates that metabolite profiling is a strong diagnostic tool to assess individual metabolic phenotypes in response to plant diversity and ecophysiological adjustment.

## Introduction

Growing awareness on accelerating rates of species loss has prompted a number of experimental studies to evaluate the relationship between plant diversity and ecosystem processes [1]. Many of these studies have shown positive effects of plant species richness on primary productivity [2], [3]. Two primary mechanisms are discussed to explain the positive diversity-productivity relationship at the community level: “sampling effects” and “complementarity effects” [4], [5], [6]. Positive sampling effects are generated by the increasing chance to include a highly productive species that dominates the community at higher diversity. Positive complementarity effects occur when differences among co-occurring species allow for resource partitioning or when facilitative interactions increase the capture of resources. Although both hypotheses propose ecologically different mechanisms, they implicitly assume that functional characteristics of particular plant species and functional differences among co-occurring plant species are responsible for the more intense exploitation of resources at higher diversity levels. Consequently, it has been observed that not species richness *per se* but also community composition strongly affects community productivity [7], [8], [9]. In particular, the presence of N_2_-fixing legumes has been identified as key component for positive plant diversity-productivity relationships in many biodiversity experiments [1]. Increasing productivity at higher plant diversity is associated with increasing canopy density and height [10], [11] and soil nutrient exploitation [12]. In addition, trophic interactions are increasingly recognized as modulating forces affecting plant species performance and community structuring. Plant diversity affects diversity and abundances of species at higher trophic levels, such as above- and belowground invertebrates or fungal pathogens [13], [14], [15]. Thus, the environment of individual plants at increasing diversity varies in multiple biotic and abiotic factors such as light and nutrient availability, plant neighbour identity and interactions with herbivores and pathogens. In spite of increasing evidence for positive plant diversity-productivity relationships, effects of plant diversity on the performance of individual species are highly variable and range from positive through neutral to negative [7], [9], [16]. So far, the mechanisms behind the variable performance of individual plant species in response to plant diversity are largely unexplained. Plants are known for their large potential in adjusting morphological and physiological characteristics in response to environmental changes. Ecophysiological adjustment must be reflected in plant metabolism and therefore can be revealed by metabolomic science [17].

**Figure 1:**
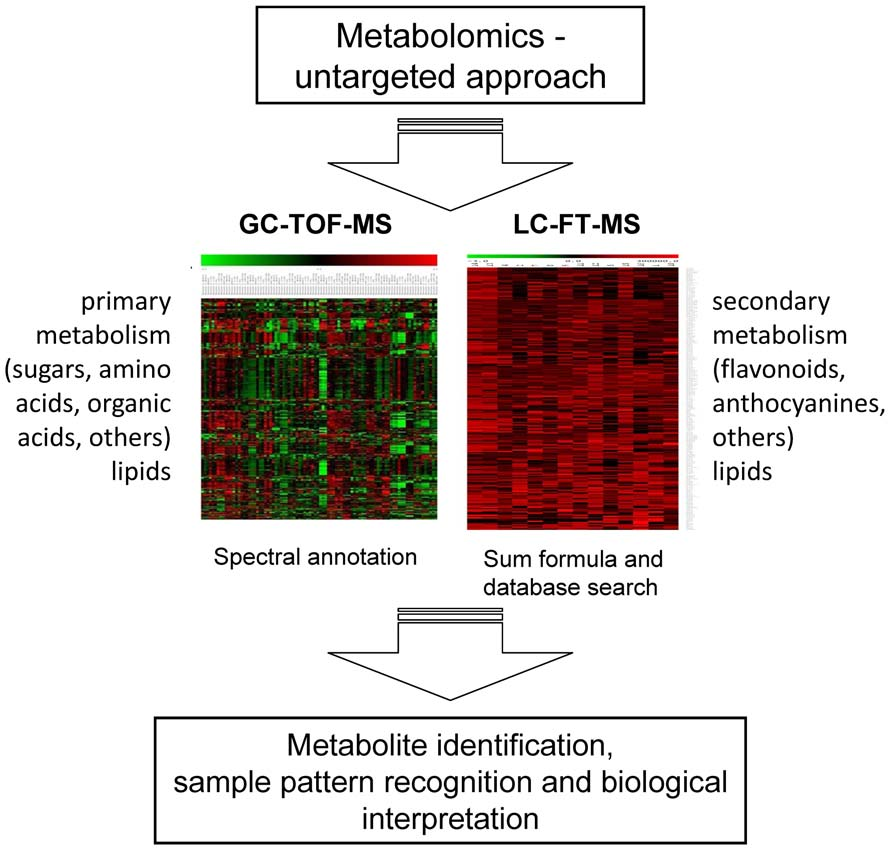
Schematic view on the techniques used to gain metabolomic coverage. A combination of GC-TOF-MS and LC-FT-MS leads to high metabolome coverage providing the basis for completely unbiased analysis (see text for more details).

Metabolomic techniques are innovative tools which have been successfully applied to analyze and interpret genetic and/or environmental perturbations, to define phenotypes of organisms and they are increasingly used in gene function annotation and systems biology [17]–[20]. In this study, we combined two fundamental and complementary techniques, gas chromatography coupled to time-of-flight-mass spectrometry (GC-TOF-MS) and ultra-performance liquid chromatography coupled to high resolution fourier transformation mass spectrometry (LC-FT-MS), to reach a higher coverage of the metabolome (Fig. 1). While GC-TOF-MS analysis basically covers metabolites from the central plant metabolism such as amino acids, sugars and organic acids [18], [21], high resolution LC-FT-MS analysis is able to measure a proportion of secondary metabolites not detectable by GC-TOF-MS (Fig. 1). Untargeted profiling allows the relative quantification of any measurable m/z-signal without requiring annotation. After statistical analysis significant changes of m/z–signals for sample classification are identified and further subjected for structural identification. This process guarantees that m/z–signals which cannot easily be annotated, however, have high statistical relevance are included in the analysis. Secondly, it enables a fast data processing using peak-picking and alignment software, which integrates m/z-signals, retention time and intensity features from several analytical runs [22]. The combination of both technologies generates data-sets, which can be regarded as a metabolomic fingerprint characterizing metabolic shifts as a consequence of plant responses to altered environmental conditions. To our knowledge, effects of plant diversity on a metabolomic scale of individual plant species have not been explored so far, especially not if the plants are sampled from their natural environment. The present study was carried out in the framework of a large biodiversity field experiment (Jena Experiment, [23]). This experiment comprises temperate grassland communities, varying in the number of different plant species (1, 2, 4, 8, 16 and 60) and the number and composition of functional groups (1 to 4; grasses, legumes, small herbs, tall herbs). For our metabolomics study we initially selected five species, which represent different plant functional groups (legumes, non-legume herbs) and differ in their growth behaviour and stature. Light (affecting the C cycle) and soil nutrients (affecting the N cycle) are the most important resources that limit plant growth in temperate grasslands [24]. Our selection criteria were based on the assumptions that, firstly, N_2_-fixing legumes are less reliant on the available soil nitrogen which decreases at higher plant diversity through a more complete exploitation of soil resources than non-legume herbs. And secondly, tall-statured species are less affected by changes in light supply through an increase in canopy density and height at higher plant diversity than small-statured species. The studied species were: *Medicago x varia* Martyn (tall-statured legume), *Knautia arvensis* (L.) J.M. Coult. (tall-statured non-legume herb), *Lotus corniculatus* L. (small-statured legume), *Bellis perennis* L. and *Leontodon autumnalis* L. (both small-statured non-legume herbs). We applied metabolomic techniques to test the following hypotheses: (i) Plant diversity in terms of species richness and legume abundance affect metabolite profiles of the model species. (ii) Metabolic responses differ among legumes and non-legumes, and tall- and small-statured herbs. (iii) Metabolic signatures and key metabolites reveal changes in resource availability at increasing plant diversity.

Metabolomics analysis revealed that plant diversity in terms of increasing species richness had significant effects on small-statured non-legume herb species indicating light and nutrient limitation, and increasing legume abundances had facilitative effects on the small-statured legume. In contrast, metabolic profiles of the tall-statured model species did not change in response to increasing plant diversity. Our study demonstrates the potential of metabolomics to identify physiological responses of plant species to changes in their natural biotic and abiotic environment and to gain insight into the mechanisms behind differential effects of plant diversity on the performance of individual species.

## Results

### Plant diversity affects individual plant metabolism

Standardized principal components analysis (PCA) of metabolite profiles derived from GC-TOF-MS data (Table 1, supporting information Fig. S1) provided evidence for differential effects of plant diversity on metabolite composition of the investigated species. The two leading PCA axes for metabolite profiles of the small-statured non-legume herbs *B. perennis* and *L. autumnalis* cumulatively explained more than 70% of the total metabolic variance of these samples grown in differently composed communities. The first PCA axis in the analysis of e.g. *B. perennis* already explained approximately 60% of the total variance allowing a full separation of the samples according to increasing species richness of the experimental plant communities. Interestingly, even though the second axis still explained 16% of the variance, it did not correlate with any of the experimental factors (Table 1) indicating large biological fluctuation of metabolite composition irrespective of plant diversity.

**Table 1:** Effects of plant diversity on metabolite composition based on GC-TOF-MS and LC-FT-MS data.

		PCA axis 1			PCA axis 2			PCA axis 3		
***Bellis perennis***	df	MS	F	p	MS	F	p	MS	F	p
Block	3	0.459	1.02	0.494	0.385	0.19	0.896	2.222	4.06	0.140
Species richness	1	6.269	**13.93**	**0.034**	1.318	0.65	0.478	0.007	0.01	0.915
Legume proportion	1	0.006	0.01	0.917	0.488	0.24	0.656	0.689	1.26	0.344
Residuals	3	0.450			2.013			0.547		
***Knautia arvensis***	df	MS	F	p	MS	F	p	MS	F	p
Block	3	0.222	0.22	0.878	0.468	0.41	0.751	0.393	0.67	0.598
Species richness	1	2.534	2.54	0.155	3.509	3.08	0.123	3.731	**6.34**	**0.040**
Legume proportion	1	2.827	2.84	0.136	0.118	0.10	0.757	3.918	**6.75**	**0.036**
Residuals	7	0.996			1.139			0.588		
***Lotus corniculatus***	df	MS	F	p	MS	F	p	MS	F	p
Block	3	0.733	1.34	0.322	0.841	0.74	0.553	0.650	0.45	0.722
Species richness	1	0.203	0.37	0.558	1.288	1.14	0.314	0.117	0.08	0.782
Legume proportion	1	7.661	**13.96**	**0.005**	0.999	0.88	0.372	0.001	0.00	0.979
Residuals	9	0.549			1.132			1.437		
***Medicago x varia***	df	MS	F	p	MS	F	p	MS	F	p
Block	3	0.414	0.50	0.696	0.819	0.73	0.572	1.867	4.23	0.063
Species richness	1	4.118	4.98	0.067	0.398	0.35	0.574	0.414	0.94	0.370
Legume proportion	1	1.675	2.02	0.205	2.377	2.11	0.197	3.337	**7.56**	**0.033**
Residuals	6	0.828			1.128			0.442		

		PCA axis 1			PCA axis 2			PCA axis 3		
***Lotus corniculatus***	df	MS	F	p	MS	F	p	MS	F	p
Block	3	1.250	2.56	0.138	1.987	2.35	0.160	0.441	0.35	0.789
Species richness	1	4.037	**8.28**	**0.024**	0.732	0.86	0.384	0.230	0.18	0.681
Legume proportion	1	1.803	3.70	0.100	0.366	0.43	0.532	2.685	2.14	0.187
Residuals	9	0.488			0.848			1.252		
***Medicago x varia***	df	MS	F	p	MS	F	p	MS	F	p
Block	3	1.446	1.63	0.296	1.412	1.69	0.284	0.797	0.66	0.609
Species richness	1	1.679	1.89	0.227	2.509	3.00	0.144	1.561	1.30	0.306
Legume proportion	1	0.550	0.62	0.446	0.073	0.09	0.779	1.050	0.88	0.392
Residuals	6	0.886			0.837			1.200		

Summary of analyses of variance (ANOVA) on the sample scores of the two leading axes of a standardized principal components analysis (PCA) of GC-TOF-MS and LC-FT-MS data (Fig. S1 and S2). Block identity, sown species richness (as log-linear term) and legume proportions (ranging from 0 when legumes were absent to 1 for pure legume communities) were fitted sequentially (type I sums of squares). Abbreviations are: df = degrees of freedom, MS = mean squares, F = F values, p = p values; significant effects are given in bold.

The two leading PCA axes for metabolite profiles of the small-statured legume *L. corniculatus* explained over 45% of the total metabolic variance. This effect is correlated to an increasing proportion of legumes in the composition of the plant communities, while species richness affects the metabolic composition of this small-statured legume only to a lower extent (Table 1). The two leading PCA axes on metabolite composition of the tall-statured species *K. arvensis* and *M. x varia* explained above 40% of total variance. However, plant diversity in terms of species richness and legume proportions did not significantly affect metabolic composition of the tall-statured model species (Table 1).

**Figure 2:**
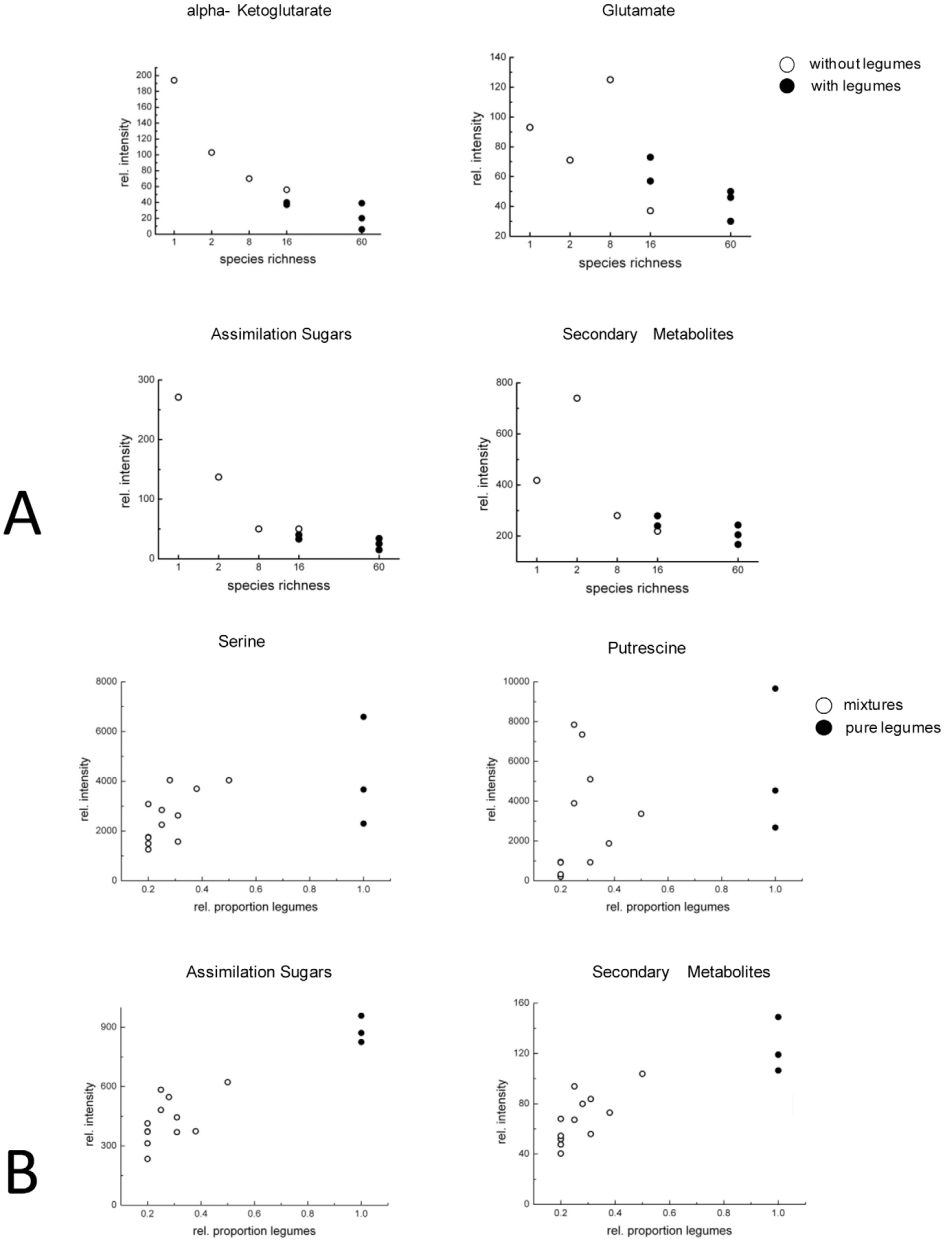
Correlation of key metabolites to species richness and legume abundance. **A** Relative intensities of key metabolites plotted against plant species richness for small-statured non-leguminous herb *Bellis perennis*. White circles symbolize plots without legumes; black circles symbolize plots with legumes. **B** Relative intensities of key metabolites plotted against relative legume proportions for the small-statured legume *Lotus corniculatus*. White circles symbolize plots with non-legumes; black circles symbolize plots where the plants were grown in monoculture or in pure legume mixtures.

Separate analyses of relative intensities of specific markers, strongly contributing to the separation of the individual plants under different growth environments, confirmed clear differences in metabolic responses to increasing plant diversity among small- and tall-statured herb species (Fig. 2, Table 2). Increasing species richness correlated with a decrease in the relative intensity of several metabolites in the small-statured non-legume herbs, especially amino acids, TCA intermediates, sugars (except for fructose and glucose), fatty acids and precursors of secondary metabolites. In contrast, *Lotus corniculatus*, the small-statured legume, showed increasing levels of amino acids, organic acids, sugars and precursors of secondary metabolites in response to increasing legume proportions (Table 2). Both species richness in case of the non-legume small-statured herbs and different legume proportions in case of the small-statured legume therefore induced significant metabolic shifts with 2–10 fold changes of individual metabolites. These results were in strong contrast to the results obtained for the tall-statured model plants *K. arvensis* and *M. x varia*, which did not show consistent metabolic changes to increasing species richness or legume proportions (Table 2). Even though some metabolites increased or decreased significantly in response to altered plant diversity, the amount of variance in the relative intensities of these metabolites was minor compared to the significant and coordinated responses observed for the small-statured plant species.

**Table 2:** Effects of plant diversity on key metabolites resulting from GC-TOF-MS data.

	*B. perennis*						*L. autumnalis*			*L.corniculatus*						*K. arvensis*						*M. x varia*					
Marker	Species richness			Leg. proportion			Species richness			Species richness			Leg. proportion			Species richness			Leg. proportion			Species richness			Leg. proportion		
	F	p		F	p		F	p		F	p		F	p		F	p		F	p		F	p		F	p	
Serine	1,52	0,305		0,09	0,788		*10,15*	*0,086*	↓	0,21	0,655		**10,59**	**0,01**	**↑**	0,3	0,6		*5,18*	*0,057*	↑	2,63	0,156		1,58	0,256	
Glycine	*57,84*	*0,005*	*↓*	0,3	0,624		–	–		–	–					–	–		–	–		*14,63*	*0,009*	*↓*	0,25	0,634	
Phenylalanine	0,06	0,817		0,74	0,452		0,93	0,437		0,43	0,53		**7,41**	**0,024**	**↑**	–	–		–	–		0,34	0,583		0,04	0,856	
Putrescine	4,03	0,429		0,43	0,522		2,03	0,29		0,25	0,627		**5,75**	**0,04**	**↑**	–	–		–	–		0,74	0,424		**9,47**	**0,022**	**↑**
beta-Alanine	*8,9*	*0,058*	↓	0,25	0,649		*18,76*	*0,049*	*↓*	0,04	0,85		*3,38*	*0,099*	↑	–	–		–	–		2,09	0,198		0,08	0,788	
Pyroglutamic acid	*19,88*	0,021	*↓*	0,45	0,552		*19,21*	*0,048*	*↓*	–	–		–	–		0	0,962		3,2	0,117		–	–		–	–	
2-Ketoglutaric acid	*28,48*	*0,013*	*↓*	0,13	0,744		5,56	0,142		–	–		–	–		–	–		–	–		1,19	0,317		0,47	0,521	
Glutamic acid	–	–		–	–		*12,53*	*0,071*	↓	–	–		–	–		–	–		–	–		**18,66**	**0,005**	**↑**	*10,56*	*0,017*	*↓*
Succinic acid	1,61	0,294		0,48	0,537		*32,56*	*0,029*	*↓*	1,38	0,27		**12,6**	**0,006**	**↑**	2,04	0,197		1	0,351		*4,06*	*0,091*	↓	1,06	0,343	
Glyceric acid	*14,01*	*0,033*	*↓*	0,01	0,94		1,08	0,407		*4,11*	*0,073*	↑	**40,88**	**<0,001**	**↑**	3,07	0,123		2,44	0,163		2,03	0,205		1,9	0,217	
Aspartic acid	*5,81*	*0,095*	↓	0,61	0,493		4,56	0,166		0,08	0,78		0,09	0,77		2,48	0,159		**9,78**	**0,017**	**↑**	**6,02**	**0,05**	**↑**	1,82	0,226	
Threonic acid	2,67	0,201		0,03	0,873		1,45	0,352		3,19	0,108		**7,64**	**0,022**	**↑**	0,66	0,443		1,41	0,274		3,34	0,117		3,26	0,121	
Shikimic acid	*9,76*	*0,052*	*↓*	0,05	0,83		0,98	0,426		–	–		–	–		**22,17**	**0,002**	**↑**	3,72	0,095		0,11	0,754		0,55	0,488	
Erythritol	*5,92*	*0,093*	*↓*	1,55	0,302		0,78	0,47		0	0,957		**17,06**	**0,026**	**↑**	0,01	0,931		0,53	0,49		1,2	0,315		2,17	0,191	
Ribitol	*10,84*	*0,046*	*↓*	0	0,949		2,12	0,283		–	–		–	–		*32,84*	*0,001*	*↓*	0,03	0,862		0,03	0,859		*4,69*	*0,073*	↓
Rhamnose	*23,78*	*0,016*	*↓*	0,04	0,85		0,39	0,596		–	–		–	–		0,24	0,636		0,01	0,944		0,68	0,44		0,03	0,867	
Fucose	*130,42*	*0,001*	*↓*	0,27	0,642		0,12	0,767		–	–		–	–		1,18	0,314		0,7	0,432		1,55	0,26		1,26	0,304	
Inositol	*11,66*	*0,042*	*↓*	0,01	0,917		2,76	0,239		2,59	0,142		0,06	0,817		0,06	0,817		0,06	0,808		1,88	0,219		3,74	0,101	
myo-Inositol	*13,9*	*0,034*	*↓*	0,64	0,484		*9,25*	*0,093*	↓	<0,01	0,997		3,07	0,114		0,71	0,429		2,38	0,167		0,37	0,564		1,27	0,303	
Trehalose	*6,44*	*0,085*	↓	0,12	0,751		1,93	0,299		*3,8*	*0,083*	↑	**11,93**	**0,007**	**↑**	–	–		–	–		–	–		–	–	
Sucrose	*8,52*	*0,062*	↓	0,45	0,552		–	–		0,14	0,715		**11,65**	**0,008**	**↑**	0,99	0,352		2,07	0,194		–	–		–	–	
Fructose	**32,88**	**0,011**	**↑**	**13,26**	**0,036**	**↑**	0,28	0,652		1,71	0,224		**18,08**	**0,002**	**↑**	0,28	0,612		*4,24*	*0,078*	↓	–	–		–	–	
Glucose	**34,17**	**0,01**	**↑**	**16,26**	**0,027**	**↑**	0,27	0,657		2,87	0,125		**18,38**	**0,002**	**↑**	0,29	0,607		**7**	**0,033**	**↑**	0,68	0,442		0,24	0,64	
Maltose	–	–		–	–		0,03	0,637		0,47	0,509		**6,64**	**0,03**	**↑**	0,15	0,71		2,72	0,143		1,07	0,341		0,41	0,546	
Dodecanoic acid	*23,12*	*0,017*	*↓*	*7,94*	*0,067*		*31,1*	*0,037*	*↓*	–	–		–	–		–	–		–	–		–	–		–	–	
Hexadecanoic acid	*19,69*	*0,021*	*↓*	0	0,976		*36,78*	*0,026*	*↓*	1,64	0,233		*4,4*	*0,065*	↑	2,47	0,16		0,18	0,68		–	–		–	–	
Octadecanoic acid	*51,12*	*0,006*	*↓*	0,27	0,639		8,3	0,102		0,78	0,401		0,03	0,86		**5,75**	**0,048**	**↑**	0,05	0,832		*4,58*	*0,076*	↑	0,45	0,528	
Caffeoylquinic acid	*7,09*	*0,076*	↓	0,02	0,897		*12,86*	*0,07*	↓	1,08	0,326		*3,49*	*0,094*	↑	–	–		–	–		–	–		–	–	
Caffeic acid	0,05	0,845		0,85	0,424		*28,76*	*0,033*	*↓*	0,23	0,644		**5,57**	**0,043**	**↑**	0,14	0,723		0,25	0,629		–	–		–	–	
p-Coumaroylquinic acid	3,65	0,152		0,02	0,896		–	–		0,01	0,924		0,04	0,84		*3,99*	*0,086*	↓	*3,68*	*0,097*	↓	**11,32**	**0,015**	**↑**	0,3	0,605	
Hydroquinone	0,91	0,411		0,09	0,782		*34,4*	*0,028*	*↓*	0,34	0,576		**26,2**	**0,001**	**↑**	*5,78*	*0,047*	*↓*	0,91	0,373		–	–		–	–	

Summary of analyses of variance (ANOVA) on relative intensities of key metabolites identified from GC-TOF-MS data for all studied species. Block identity, sown species richness (as log-linear term) and legume proportions (ranging from 0 when legumes were absent to 1 for pure legume communities) were fitted sequentially (type I sums of squares). For abbreviation see Table 1. Significant effects of increasing species richness and increasing legume proportions were marked in bold (increase: ↑) and italics (decrease: ↓).

### Plant diversity affects both primary and secondary metabolism

Next to the in depth analysis of primary metabolites by GC-TOF-MS we subjected samples of two legume species, the small-statured *L. corniculatus* and the tall-statured *M. x varia*, to LC-FT-MS analysis. This technique, in contrast to the GC-TOF-MS, detects semi-polar compounds, representing a significant proportion of secondary metabolites such as flavonoids, alkaloids, indolic compounds, phenylpropanoids as well as some abundant polar lipids.

In analogy to the PCA analysis of the GC-TOF-MS data, the PCA analysis of the LC-FT-MS data showed similar responses of the small- and the tall-statured plant species to plant diversity. Species richness correlating with decreasing legume proportions explained sample separation based on LC-FT-MS data on the first PCA axis for *L. corniculatus*, but did not explain a significant proportion of variation in LC-FT-MS data of *M. x varia* (Table 1, supporting information Fig. S2).

**Figure 3:**
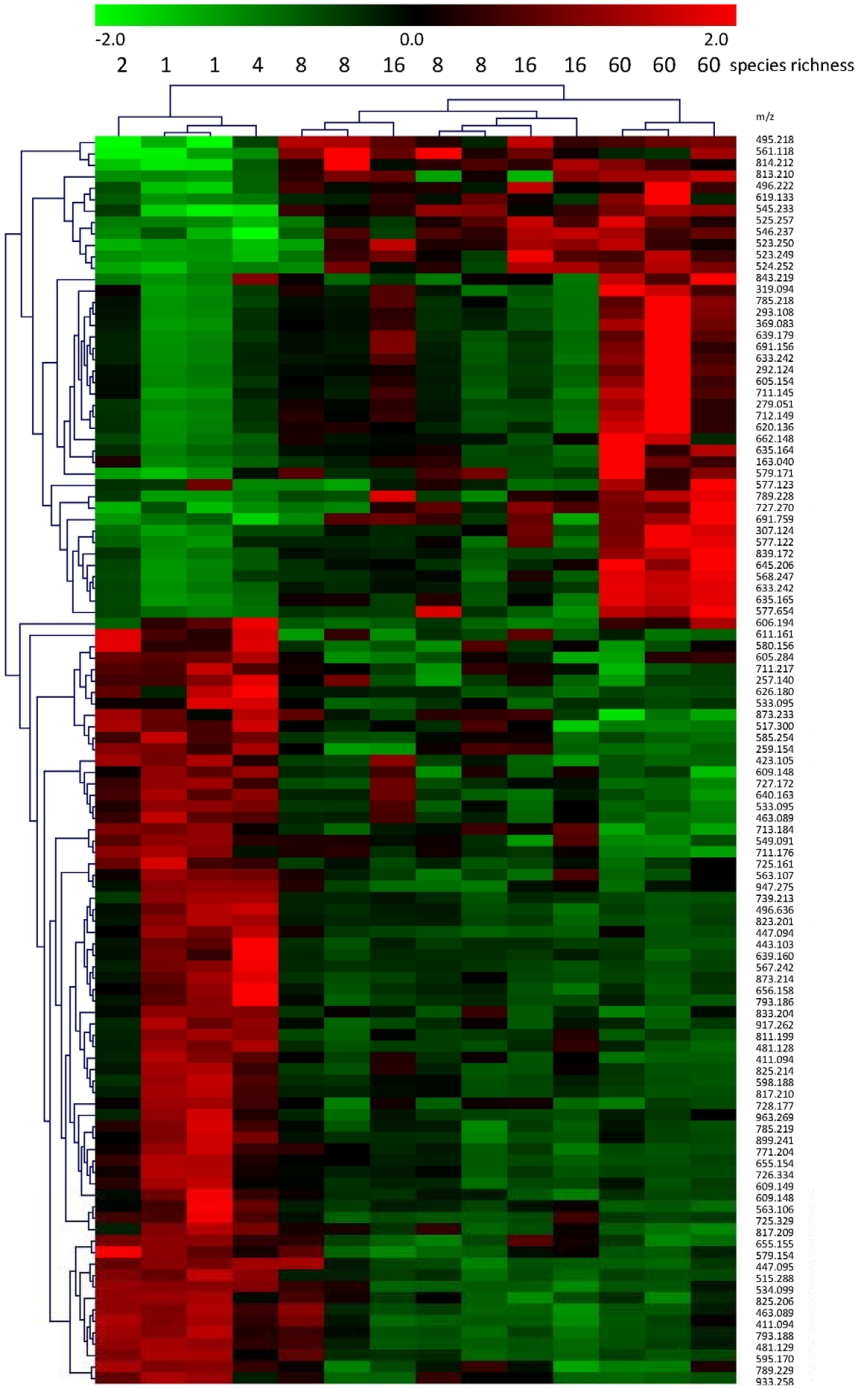
Significant changes in secondary metabolites of *L. corniculatus* in response to plant species richness. A color-coded display of significant changes in the metabolite level (relative changes; data are standardized) is given where green indicates a down-regulation and red indicates an up-regulation; exact masses of possible metabolites are shown on the right side. Test on significant differences among three groups of samples resulting from cluster analysis were performed with Kruskal-Wallis tests (selected FDR limit = 0.05; Bonferroni adjustment); clustering was based on Euclidean distance and average linkage. Group 1 (n = 4) summarizes communities with 1 to 4 species; group 2 (n = 4) are communities with 8 species; group 3 (n = 6) are communities with 16 and 60 species. A pre filtered data matrix was applied to the test and resulted in 109 significantly changed m/z and 725 non-significantly changed m/z.

Samples of *L. corniculatus* could be classified into three groups based on the PCA data, comprising plant communities with 1 to 4 species, 8 species and 16 to 60 species (n≥4 plots per group). To test for the robustness of these groups each metabolic feature was independently tested by a Kruskal-Wallis test. The visualization of this data clearly shows positive and negative species richness-metabolite correlations (Fig. 3). Over 109 of these spectroscopic features significantly differed between sample groups collected in plant communities with different species richness. In contrast to the GC-TOF-MS data, the metabolic features from the LC-FT-MS are not represented by a distinct metabolite but through an accurate mass to charge-ratio (m/z) (better than 2ppm), which allows for a preliminary annotation via database search [25]. The accurate mass to charge-ratios were searched against a number of comprehensive metabolite databases using an in-house-developed database search tool (GoBioSpace, http://gmd.mpimp-golm.mpg.de/webservices/wsGoBioSpace.asmx).

In Table S1, supporting information, a list of matching elemental compositions related to possible metabolite candidates is shown exhibiting a significant correlation to plant diversity. Many measured metabolic features from the different samples, even though they correlated strongly with species richness, did not provide a match to any database showing that a large number of biologically relevant compounds are thus far un-described.

## Discussion

The aim of this study was to test if metabolomic techniques are useful diagnostic tools in environmental studies to characterize how plant species respond to multiple changes in their biotic and abiotic environment at increasing plant diversity. Positive effects of plant diversity on primary productivity correlating with increasing canopy density and height have been reported from biodiversity experiments, including the Jena Experiment [9], [10]. Interactions between legumes and non-legumes have been repeatedly identified as a key mechanism for positive diversity-productivity relationships [1]. These relationships might be explained by improved nitrogen availability in communities containing N_2_-fixing legumes, promoting complementary resource use and facilitation [26]. Nevertheless, plant diversity has highly variable effects on the performance of individual plant species. So far, it remained unresolved whether the more complete use of available resources at increasing plant diversity is associated with a resource limitation of particular plant species which would offer a mechanistic explanation for their decreasing performance. To test this hypothesis, we selected a set of species varying in their ability to fix atmospheric nitrogen (legumes vs. non-legumes) and growth height (tall-statured vs. small-statured plants) and investigated changes in their metabolic profiles in response to increasing species richness and legume proportions in the Jena Experiment.

### Metabolic responses of small-statured species to increasing plant diversity

**Figure 4:**
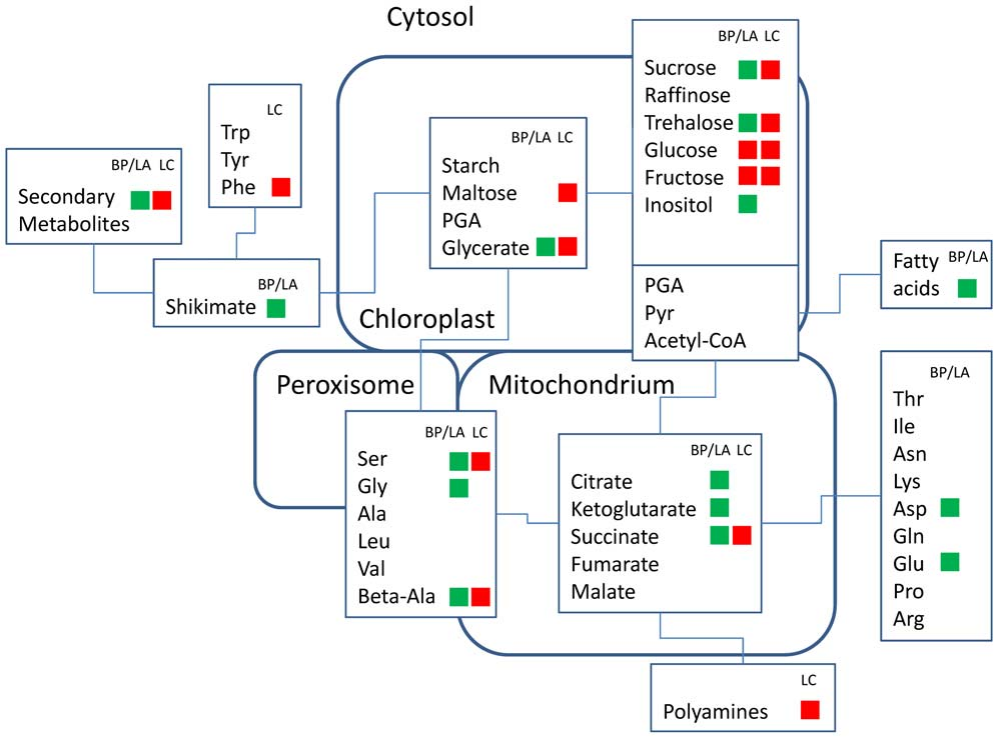
Metabolic signatures of the primary metabolism of small-statured plant species in response to plant diversity. Significant changes were symbolized via heat map (red = relative increase in response to plant diversity; green = relative decrease in response to plant diversity). BE/LA corresponds to relative metabolite concentration changes of *Bellis perennis* and *Leontodon autumnalis* in response to species richness. LC corresponds to relative metabolite concentration changes of *Lotus corniculatus* in response to increasing proportions of legumes.

Untargeted metabolite profiling approaches revealed that plant diversity correlated significantly with variation in metabolic patterns of *B. perennis*, *L. autumnalis* and *L. corniculatus*. Increasing species richness or legume proportions led to significant metabolic shifts of their primary and secondary metabolism summarized in metabolic signatures (Fig. 4). In the non-leguminous small-statured model species, the reduced N and C intermediates of the primary metabolism and related pathways (fatty acids, secondary metabolites) indicated a resource limitation with increasing plant species richness. Plants must achieve a balance between carbon assimilation, carbon storage and growth. Assimilation sugars serve as a link from source to sink activity to produce amino acids or secondary metabolites from carbon bodies. As a consequence, low carbohydrate concentrations indicate a source limitation (low rate of C supply) which correlates with reduced levels of organic acids [27], [28]. Thus, it is likely that increasing canopy density and height at increasing productivity levels in more diverse plant communities induced a C-limitation in the investigated small-statured non legumes. Increasing species richness was partially confounded with a higher probability to include legumes (Fig. 2). Although increasing species richness and legume proportions correlated with a decrease of the assimilated sugars suggesting a limitation in C assimilation (photosynthesis), decreasing amino acid levels indicated a nitrogen limitation (low rate of N supply) even in plots with larger legume proportions. These results are in line with previous analyses of carbohydrate and nitrate concentrations in leaves of the subordinate grass species *Lolium perenne* L. which indicated a C- and N-limitation at increasing species richness and lower legume proportions [29].

In contrast, the small-statured legume *L. corniculatus* had significantly increased levels of C and N intermediates of the primary metabolism in plant communities with proportionally more legumes. In particular, the increased levels of amino acids, organic acids and assimilation sugars imply that the small-statured legume *L. corniculatus* benefits from the presence of other legumes in legume-rich assemblages. Strong effects of legumes on ecosystem processes are known from many biodiversity experiments [1]. Legumes improve soil N availability for neighbouring plants through rhizodeposition, mycorrhizal links and decomposition of their litter with low C∶N ratios. However, legumes may suppress their energy-consuming symbiotic N_2_ fixation at high soil N availability. In the Jena Experiment N_2_ fixation of legumes increased with plant diversity and increasing proportions of non-legumes in mixtures [30]. Decreasing levels of C and N intermediates of the primary metabolism in plant communities with lower legume proportions suggested large effects of non-leguminous species on the metabolism of *L. corniculatus* which indicates facilitating effects of co-occurring legumes. In addition, our results indicated strong effects of species richness on the secondary metabolism of the legume *L. corniculatus* (Fig. 3). Because of the large capacity of the LC-FT-MS technique [31] high metabolome coverage can be assumed by these complementary data. However, the identification of metabolites solely from accurate mass-to-charge-ratio is severely hampered [31], [32]. Future studies are necessary to elucidate the chemical structures of significantly affected secondary metabolites in our data set.

In comparison, the primary and secondary metabolism of the tall-statured species *K. arvensis* and *M. x varia* was weakly affected by increasing plant diversity. Obviously, the tall-statured model species did not suffer from resource limitation in more diverse plant communities.

### Species-specific responses to environmental changes with increasing plant diversity

It is well known that variation in morphological and physiological properties enables plant species to survive and compete successfully in a wide range of environmental conditions. Nevertheless, in temperate grasslands only few tall-growing species usually produce a major proportion of community biomass, while a large number of subordinate species achieve a low biomass production [33], [34]. Investigating physiological and morphological characteristics thus provides important information to assess which mechanisms are responsible for differences in species competitive ability and abundances in different biotic and abiotic environments. Previous studies have shown that the tall-statured species *K. arvensis* and *M. x varia* as well as the small-statured herb *B. perennis* achieve a higher biomass in increased plant diversity treatments than in monocultures in the Jena Experiment [9], [30], [35]. In contrast, the small-statured species *L. autumnalis* and *L. corniculatus* usually are less productive in mixtures than expected from their monocultures [9], [36]. *Leontodon autumnalis* even went extinct in several plots of higher species richness and could only be sampled in few plots in the current study (Table S2, supporting information). Morphological variation at the whole-shoot and leaf-level indicating typical shade-avoidance or shade–tolerance strategies have been previously shown to be larger in small-statured subordinate species than in tall-statured species at increasing plant diversity [35], [36]. Nevertheless, such morphological responses of small-statured species do not necessarily have an adaptive value if they do not improve whole-plant energy capture [37]. The metabolomic screening showed that primary and secondary metabolite levels of small-statured species underlie strong shifts which indicated C and N limitation of small-statured species at increasing plant diversity. Thus increasing resource limitation appears to be responsible for decreasing performance of these species in response to increasing plant diversity, while plant diversity had little effects on metabolic composition of the tall-statured species.

### Conclusions

In summary, our study exemplifies that environmental changes, as mediated by variation in light or nutrient availability, induce physiological adaptations of individual plant species to increasing plant diversity. The application of modern metabolomic techniques reveals specific physiological responses of plant species to changing biotic interactions at different trophic levels. However, further method development especially in the field of structure elucidation of complex secondary metabolites is necessary to exploit the full potential of metabolomics in environmental studies. Thus, metabolomic techniques are an important diagnostic tool to verify plant species physiological responses to environmental changes and to get further insights into the mechanisms behind biodiversity-ecosystem functioning relationships. In the future, these metabolomic studies will strengthen our understanding of the specific genotype-environmental phenotype-relationship, especially in combination with next generation sequencing (NGS) techniques. Further, it can be expected that metabolomic studies will be helpful to investigate epigenetic adaptation processes of species in their environment.

## Materials and Methods

### Experimental design

The Jena Experiment is a large biodiversity experiment established on a former agricultural field in 2002. The experimental site is located in the floodplain of the river Saale in Jena (Thuringia, Germany, 50°55′N, 11°35′E, 130 m a.s.l.). Mean annual air temperature is 9.3°C, and average annual precipitation is 587 mm in the area around Jena [38]. Plant communities were created based on a species pool of 60 plant species common to Central European Arrhenatherion grasslands (Molinio-Arrhenatheretea meadows, [23], [39]). Plant species were assigned to four functional groups (FG): grasses (16 species), legumes (12 species), small herbs (12 species), and tall herbs (20 species). The experimental design varies the factors plant species richness (1, 2, 4, 8, and 16 species) near-orthogonal to the number of plant functional groups (1, 2, 3, and 4) and comprises 16 replicates per species-richness level except for 14 replicates at the 16-species level (because pure legume or small herb mixtures were not possible). Species for each mixture were chosen randomly with replacement. In addition, four plots with a 60-species mixture were established resulting in a total of 82 plots of 20×20 m size. Furthermore, each species was grown in a monoculture plot of 3.5×3.5 m size. The experimental field was divided into four blocks, taking into account a gradient in soil characteristics with growing distance to the riverside. Plots were sown with a constant total density of 1000 viable seeds per m^2^, reducing sowing densities per species proportional to the number of species in the mixture in a substitutive design. Further details on the experimental design are given in [23]. Plots were weeded regularly to maintain sown species combinations. They were mown twice a year, early in June and September, corresponding to the typical management of extensively used hay meadows in the region. Plots did not receive any fertilization.

### Studied species and plant sampling

Five plant species representing different plant functional groups – legumes and non-leguminous herbs – were selected for metabolomic screening. The non-leguminous herbs *Bellis perennis* (Asteraceae) and *Leontodon autumnalis* (Asteraceae) are small-statured rosette plants (growth height 5 and 15 cm respectively) with an adventitious root system. The non-leguminous herb *Knautia arvensis* (Dipsacaceae) grows with a semi-rosette reaching a growth height of 30–80 cm and has a tap root of 80–150 cm depth. *Lotus corniculatus* (Fabaceae) and *Medicago x varia* (Fabaceae) grow with a tap root of 90–150 cm depth. *Lotus corniculatus* has a short growth height of 20–40 cm compared to *M. x varia* with a growth height of 60 cm [40]. Table S2, supporting information, gives an overview on the occurrence and replication of the studied species in the experimental plots.

Material of approximately 15 fully expanded leaves from at least three different plant individuals per plot and species was collected at midday (12:00–13:30) on 23 May 2008 at estimated maximum canopy development before first mowing. The plant material was immediately snap-frozen in liquid nitrogen and stored in a freezer (−80°C) until sample processing.

### GC-TOF-MS Extraction and derivatisation

Approximately 10–15 mg frozen plant material was homogenized in a 2 mL Eppendorf tube, and 1 ml metabolite extraction buffer (methanol/chloroform/water; 2.5∶1∶0.5; v/v/v) and 10 µl internal standard D-sorbitol-^13^C_6_ (200 µg/mL in H_2_O) was added subsequently. The organic phases were fractionated into a polar and lipophilic phase by addition of 0.5 mL ddH_2_O and centrifugation. The upper polar phases were used for GC-TOF-MS analysis and dried completely in a speed-vac concentrator. Pellets were re-dissolved and derivatized for 90 min at 30°C (20 µl of 40 mg/mL methoxyamine hydrochloride in dry pyridine) followed by a 30 min treatment at 37°C (with 90 µl MSTFA). In every sample, 3 µL of the retention time index marker (n-alkanes ranging from C10 to C36, each 200 µg /ml in pyridine) was spiked to determine the retention time index (RI) [18].

### GC-TOF-MS settings

For plant metabolite profiling gas chromatography (GC) coupled to a LECO® Pegasus IV TOF (Leco Corp Inc., St. Joseph, USA) mass analyzer (GC-TOF-MS) was used according to [18]. Analysis was performed on an HP6890 gas chromatograph with deactivated standard spit/splitless liners containing glasswool (Agilent, Böblingen, Germany). Sample volumes of 1 µl were injected in splitless mode at 230°C injector temperature. GC was operated on a VF-5ms capillary column, 30 m long, 0.25-mm inner diameter, 25 µm film thickness (VARIAN, Palo Alto, USA), at constant flow of 1 mL/min helium. The temperature program started with 1 min isocratic at 70°C, followed by temperature ramping at 9°C/min and a final temperature of 350°C which was held for 5 min. Scan rates of at 20 s^−1^ and mass ranges of 70–600 Da were used.

### GC-TOF-MS data mining

First, the raw-data were analyzed with the ChromaTof® software from LECO®, which performed the deconvolution of all mass spectra, built mass-spectral correction for co-eluting metabolites, calculated the RI's, and identified a suitable fragment mass-to-charge ratio for selective quantification. The obtained data were analyzed by defining a reference chromatogram with the maximum number of detected peaks over a signal/noise threshold of 50. All chromatograms were matched against this reference chromatogram with a minimum match factor of 800. Compounds were annotated by retention index and mass spectra comparison to a user-defined spectra library. Selected fragment ions specific for each individual metabolite were used for peak area quantification. Each compound was normalized by the peak area from the internal standard and by the fresh weight of each sample. In a second approach we used the TagFinder for data mining [41]. The GC-TOF-MS measurements generated about 20.000 so-called mass spectral tags (MST) per run [42], which were combined into chromatographic time groups resulting in an output of approximately 600 potential compounds.

### UPLC-NanoMate-FT-ICR MS measurement - Extraction

50 mg of frozen plant material was homogenized in a 2 mL Eppendorf tube, and 1 ml methanol (100%) containing two internal standards (1µg/mL ampicillin and 1µg/mL chloramphenicol) were added. After vortexing and centrifugation the soluble metabolites were dried in a speed-vac concentrator. The dried residue was re-dissolved in 100 µL 50% methanol before measurement.

### UPLC-FT-ICR-MS settings

UPLC separation was performed with a Waters Acquity UPLC system (Waters, Mildford, MA, USA), using a HSS T3 C_18_ reversed phase column (100×2.1 mm i.d. 1.8 µm particle size, Waters) at a temperature of 40°C. The mobile phases consisted of 0.1% formic acid in water (Solvent A), and 0.1% formic acid in acetonitrile (Solvent B). The flow rate of the mobile phase was 400 µL/min and 2µL sample were loaded per injection. The following gradient profile was applied: After 1 min of isocratic run at 99% A a linear 12 min gradient was applied to 65% A immediately followed by an 1.5 min gradient to 30% A and a 1 min gradient to 1% A. Then a 1.5 min isocratic period at 1% A followed before switching back to 99% A to re-equilibrate the column for 2.5 min before to the next sample was injected. The UPLC was connected to the FT-ICR via a TriVersa NanoMate (Advion, Ithaca, NY, USA). The UPLC flow rate at 400 µL/min was split 1∶1000 with a T-Valve (Advion). One tenth of a percent (400nL/min) was directly loaded to the FT-ICR MS, while 99.9% were discarded. The sample was infused into the MS via a nanospray Chip (Type A, Advion), by applying a voltage of 1.8 kV in the positive and 1.9 kV in the negative ionization mode. Spray sensing was used between min 1 and 17 of the UPLC gradient. The mass spectra were acquired using the LTQ FT-ICR-Ultra mass spectrometer (Thermo-Fisher, Bremen, Germany). The spectra were recorded using full scan mode, covering a mass range from m/z 100–1300. Resolution was set to 50,000, and maximum loading time for the ICR cell was set to 500ms. The transfer capillary temperature was set to 200°C and the MS spectra were recorded from min 1 to 17 of the UPLC gradient.

### UPLC-FT-ICR-MS data mining

The global metabolic profiling approach via UPLC-FT-ICR-MS considers every measured m/z - signal as a possible metabolite (feature) and is in first place not restricted on mass spectral and chromatographic comparison of authentic reference substances [25].

Molecular masses, retention time and associated peak intensities were extracted from the raw files using the SIEVE software (Version 2.0, Thermo-Fisher). The mass and retention time lists were used for searches against the ChemSpider database, employing the in-house developed database search tool GoBioSpace (http://gmd.mpimp-golm.mpg.de/webservices/wsGoBioSpace.asmx). This tool was realized using a Microsoft SQL Server 2005 as the relational database backend for storing chemical sum formula with appropriate source tagged annotations (names, synonyms, cross references, etc). Algorithms for formula parsing and isotopic correct mass calculation were implemented as User-defined Types using the Common Language Runtime (CLR) net framework, the C# programming language, and Microsoft Visual Studio 2005. The search criteria, which can be restricted to a mass error of between 0.1–100 ppm, were set to 2 ppm and only chemical formulas containing the elements C, H, N, O, P or S were allowed for the final result table.

The result files, including the database annotations of each mass, associated chemical formula, retention time, m/z value, compound ID, and possible substance names, were exported as text files. Data visualization was performed using TIGR MeV 4.1 software. The content of text files was sorted and filtered either directly in the GoBioSpace search tool or by using Microsoft Access® (Access 2007, Microsoft). All other spectra manipulations and peak extractions were performed using Xcalibur (Version 2.06, Thermo Fischer).

The list of chemical formulas calculated in GoBioSpace still overestimated the number of truly distinct biological compounds present in the investigated plant material. Many compounds were annotated with more than one chemical sum formula and amplified the number of “true” database hits.

### Data analysis and statistics

Relative intensities of metabolites detected by GC-TOF-MS were analysed separately with general linear models with sequential sum of squares (type I sums of squares). Although the Jena Experiment has a near-orthogonal design [23], individual species were randomly assigned to the experimental mixtures and are not evenly distributed among the levels of species and functional group richness or the experimental blocks. Based on the hypotheses of our study, our analyses focused on effects of species richness and legume abundance. Model terms were fitted in the following sequence: firstly, block was entered to account for variance caused by the gradient of edaphic conditions in the field site and effects of the block-wise organized management (weeding, mowing) and sample collection. In the following steps the number of sown species (as log-linear term) and sown proportions of legumes (ranging from 0 when legumes were absent to 1 for pure legume communities) were fitted. In a second series of models fitting order of the species richness and the legume term were changed. The order of terms affected the outcome of data analyses only in a few cases and results are only mentioned when necessary. Only species richness effects were tested in analyses of *L. autumnalis* because of low number of replicates.

In addition, standardized principal components analysis (PCA) was applied to explore differences in metabolic composition based on GC-TOF-MS data for all studied species and LC-FT-MS data for legume species. The scores, i.e. the relative positions of samples on the two leading PCA axes, were also analysed with general linear models as described above (except for *L. autumnalis* because of low sample number) to assess the significance of the experimental factors on overall metabolic composition. Data analyses were performed with the statistical software R (Version 2.6.2, R Development Core Team 2007, http://www.R-project.org) and CANOCO 4.5. If necessary, variables were log-transformed to meet the assumptions of ANOVA:

To visualise metabolic changes in the LC-FT-MS profiles of *L. corniculatus* in response to species diversity, cluster and variance analysis was combined with the TIGR MeV software version 4.1, whereby each metabolite is separately tested by the software. Clustering was based on Euclidean distance and average linkage. The variance of each metabolite was analyzed via Kruskal-Wallis tests with Bonferroni adjustment between three groups of samples. Metabolite data matrices were standardized via a z-transformation.

## Supporting Information

Table S1 Database matches for secondary metabolites affected by increasing plant species richness. Formulas were calculated with exact masses with 2ppm accuracy with the tool GoBioSpace (\\HOMES\Exchange_MPI$\AG_Bioinformatics\JaHu\GoBioSpace).
(0.06 MB DOC)

Table S2 Number of plots per species-richness level where plant material of the five studied species was collected. Plots are classified in community without/with legumes for non-leguminous herb species, and communities with/without non-legumes for legume species. The number of plots in which the original seed mixtures contained the studied species is given in parentheses.
(0.03 MB DOC)

Figure S1 Biplots of standardized principal components analysis (PCA; first vs. second axis) based on GC-TOF-MS metabolite data analysed for (a) Medicago x varia, (b) Lotus corniculatus, (c) Knautia arvensis, (d) Bellis perennis, and (e) Leontodon autumnalis. Labels indicate the number of species in the assemblages where species were sampled (1sp = monoculture, 2sp = 2-species mixture, 4sp = 4-species mixture, 8sp = 8-species mixture, 16sp = 16-species mixture, 60sp = 60-species mixture). Symbols indicate: white squares = investigated legumes grown in combination with non-legumes; black squares = investigated legumes grown in monoculture or in pure legume mixtures; black circles = investigated non-leguminous herbs grown in combination with legumes; white circles = investigated non-leguminous herbs grown in monoculture or in pure non-legume mixtures.
(0.04 MB TIF)

Figure S2 Biplots of standardized principal components analysis (PCA; first vs. second axis) based on LC-FT-MS metabolite data analysed for (a) Medicago x varia, and (b) Lotus corniculatus. Labels indicate the sown number of species in the assemblages where species were sampled (abbreviations see also legend of Fig. S1). Symbols indicate: white squares = assemblages where the studied legumes grew in combination with non-legumes; black squares = assemblages where the studied legumes grew in monoculture or in pure legume mixtures.
(0.03 MB TIF)

## References

[1] Hooper DU, Chapin FS, Ewel JJ, Hector A, Inchausti P (2005). Effects of biodiversity on ecosystem functioning: A consensus of current knowledge.. Ecological Monographs.

[2] Balvanera P, Pfisterer AB, Buchmann N, He JS, Nakashizuka T (2006). Quantifying the evidence for biodiversity effects on ecosystem functioning and services.. Ecology Letters.

[3] Cardinale BJ, Wright JP, Cadotte MW, Carroll IT, Hector A (2007). Impacts of plant diversity on biomass production increase through time because of species complementarity.. Proceedings of the National Academy of Sciences of the United States of America.

[4] Aarssen LW (1997). High productivity in grassland ecosystems: effected by species diversity or productive species?. Oikos.

[5] Huston MA (1997). Hidden treatments in ecological experiments: Re-evaluating the ecosystem function of biodiversity.. Oecologia.

[6] Tilman D, Knops J, Wedin D, Reich P, Ritchie M (1997). The influence of functional diversity and composition on ecosystem processes.. Science.

[7] HilleRisLambers J, Harpole WS, Tilman D, Knops J, Reich PB (2004). Mechanisms responsible for the positive diversity-productivity relationship in Minnesota grasslands.. Ecology Letters.

[8] Spehn EM, Hector A, Joshi J, Scherer-Lorenzen M, Schmid B (2005). Ecosystem effects of biodiversity manipulations in European grasslands.. Ecological Monographs.

[9] Marquard E, Weigelt A, Roscher C, Gubsch M, Lipowsky A (2009). Positive biodiversity-productivity relationship due to increased plant density.. Journal of Ecology.

[10] Spehn EM, Joshi J, Schmid B, Diemer M, Korner C (2000). Above-ground resource use increases with plant species richness in experimental grassland ecosystems.. Functional Ecology.

[11] Lorentzen S, Roscher C, Schumacher J, Schulze ED, Schmid B (2008). Species richness and identity affect the use of aboveground space in experimental grasslands.. Perspectives in Plant Ecology Evolution and Systematics.

[12] Oelmann Y, Kreutziger Y, Temperton VM, Buchmann N, Roscher C (2007). Nitrogen and phosphorus budgets in experimental grasslands of variable diversity.. Journal of Environmental Quality.

[13] Knops JMH, Tilman D, Haddad NM, Naeem S, Mitchell CE (1999). Effects of plant species richness on invasion dynamics, disease outbreaks, insect abundances and diversity.. Ecology Letters.

[14] Koricheva J, Mulder CPH, Schmid B, Joshi J, Huss-Danell K (2000). Numerical responses of different trophic groups of invertebrates to manipulations of plant diversity in grasslands.. Oecologia.

[15] Mitchell CE, Tilman D, Groth JV (2002). Effects of grassland plant species diversity, abundance, and composition on foliar fungal disease.. Ecology.

[16] Roscher C, Schumacher J, Weisser WW, Schmid B, Schulze ED (2007). Detecting the role of individual species for overyielding in experimental grassland communities composed of potentially dominant species.. Oecologia.

[17] Weckwerth W (2003). Metabolomics in systems biology.. Annu Rev Plant Biol.

[18] Morgenthal K, Wienkoop S, Scholz M, Selbig J, Weckwerth W (2005). Correlative GC-TOF-MS based metabolite profiling and LC-MS based protein profiling reveal time-related systemic regulation of metabolite-protein networks and improve pattern recognition for multiple biomarker selection.. Metabolomics.

[19] Fiehn O, Kopka J, Dormann P, Altmann T, Trethewey RN (2000). Metabolite profiling for plant functional genomics.. Nature Biotechnology.

[20] Viant MR (2008). Recent developments in environmental metabolomics.. Molecular Biosystems.

[21] Weckwerth W, Wenzel K, Fiehn O (2004). Process for the integrated extraction identification, and quantification of metabolites, proteins and RNA to reveal their co-regulation in biochemical networks.. Proteomics.

[22] Hoehenwarter W, van Dongen JT, Wienkoop S, Steinfath M, Humme J (2008). A rapid approach for phenotype-screening and database independent detection of cSNP/protein polymorphism using mass accuracy precursor alignment.. Proteomics.

[23] Roscher C, Schumacher J, Baade J, Wilcke W, Gleixner G (2004). The role of biodiversity for element cycling and trophic interactions: an experimental approach in a grassland community.. Basic and Applied Ecology.

[24] Grime JP (1973). Competitive Exclusion in Herbaceous Vegetation.. Nature.

[25] Giavalisco P, Kohl K, Hummel J, Seiwert B, Willmitzer L (2009). C-13 Isotope-Labeled Metabolomes Allowing for Improved Compound Annotation and Relative Quantification in Liquid Chromatography-Mass Spectrometry-based Metabolomic Research.. Analytical Chemistry.

[26] Temperton VM, Mwangi PN, Scherer-Lorenzen M, Schmid B, Buchmann N (2007). Positive interactions between nitrogen-fixing legumes and four different neighbouring species in a biodiversity experiment.. Oecologia.

[27] Stitt M, Schulze D (1994). Does Rubisco Control the Rate of Photosynthesis and Plant- Growth - an Exercise in Molecular Ecophysiology.. Plant Cell and Environment.

[28] Stitt M, Krapp A (1999). The interaction between elevated carbon dioxide and nitrogen nutrition: the physiological and molecular background.. Plant Cell and Environment.

[29] Roscher C, Kutsch W, Schulze E-D (2010). Light and nitrogen competition limit Lolium perenne in experimental grasslands of increasing plant diversity.. Plant Biology.

[30] Roscher C, Buchmann N, Schmid B, Weigelt A, Schulze E-D (2010). Legume species differ in the responses of their functional traits to plant diversity.. Oecologia.

[31] Giavalisco P, Hummel J, Lisec J, Inostroza AC, Catchpole G (2008). High-Resolution Direct Infusion-Based Mass Spectrometry in Combination with Whole C-13 Metabolome Isotope Labeling Allows Unambiguous Assignment of Chemical Sum Formulas.. Analytical Chemistry.

[32] Kind T, Fiehn O (2006). Metabolomic database annotations via query of elemental compositions: Mass accuracy is insufficient even at less than 1 ppm.. BMC Bioinformatics.

[33] Gaudet CL, Keddy PA (1988). A Comparative Approach to Predicting Competitive Ability from Plant Traits.. Nature.

[34] Grime JP (1998). Benefits of plant diversity to ecosystems: immediate, filter and founder effects.. Journal of Ecology.

[35] Dassler A, Roscher C, Temperton VM, Schumacher J, Schulze ED (2008). Adaptive survival mechanisms and growth limitations of small-stature herb species across a plant diversity gradient.. Plant Biology.

[36] Thein S, Roscher C, Schulze ED (2008). Effects of trait plasticity on aboveground biomass production depend on species identity in experimental grasslands.. Basic and Applied Ecology.

[37] Weinig C (2000). Limits to adaptive plasticity: Temperature and photoperiod influence shade-avoidance responses.. American Journal of Botany.

[38] Kluge G, Müller-Westermeier G (2000).

[39] Ellenberg H (1988).

[40] Rothmaler R, Jäger EJ, Werner K (2002). Exkursionsflora von Deutschland. Bd. 4. Kritischer Band.. 9th ed., Spektrum.

[41] Luedemann A, Strassburg K, Erban A, Kopka J (2008). TagFinder for the quantitative analysis of gas chromatography - mass spectrometry (GC-MS)-based metabolite profiling experiments.. Bioinformatics.

[42] Erban A, Schauer N, Fernie AR, Kopka J (2007). Nonsupervised construction and application of mass spectral and retention time index libraries from time-of-flight gas chromatography-mass spectrometry metabolite profiles.. Methods Mol Biol.

