# Supplementary material for: Metabolomics Unravel Contrasting Effects of Biodiversity on the Performance of Individual Plant Species
Source: PLoS One. 2010 Sep 7;5(9):e12569. doi: 10.1371/journal.pone.0012569 (PMC2935349; doi:10.1371/journal.pone.0012569)
Supplement: Table S1 — Database matches for secondary metabolites affected by increasing plant species richness. Formulas were calculated with exact masses with 2ppm accuracy with the tool GoBioSpace (\\HOMES\Exchange_MPI$\AG_Bioinformatics\JaHu\GoBioSpace). (0.06 MB DOC) [file pone.0012569.s001.doc]

Table S1

| **Metabolite** | | | **ANOVA** | | | **correlation** | **potential formulas by 2ppm** | | **database entries** | | | |
| --- | --- | --- | --- | --- | --- | --- | --- | --- | --- | --- | --- | --- |
| **mode** | **m/z** | **time** | **F-Test** | **groups** | **p-value** | **R2** |
| neg | **645,2066** | 8,95 | 69,81 | 4,00 | 5.22E-07 | **0.95** | 3,00 | **C38H34N2O4P2** | ChemSpider ID: 93345051 |  |  |  |
|  |  |  |  |  |  |  |  | **C31H32N8O4S2** | ChemSpider ID: 12932623 |  |  |  |
|  |  |  |  |  |  |  |  | **C26H44O12S3** | MJ088232 | MJ088233 |  |  |
|  |  |  |  |  |  |  |  |  |  |  |  |  |
| neg | **568,2473** | 4,99 | 49,35 | 4,00 | 2.67E-06 | **0.92** | 3,00 | **C30H37N3O6S** | ChemSpider ID: 414946 | ChemSpider ID: 415024 | ChemSpider ID: 449069 | ChemSpider ID: 2467031 |
|  |  |  |  |  |  |  |  | **C38H33NO4** | ChemSpider ID: 490436 | ChemSpider ID: 4102285 | ChemSpider ID: 4158108 | ChemSpider ID: 8113523 |
|  |  |  |  |  |  |  |  | **C22H41N5O8S2** | CID: 22362094 |  |  |  |
|  |  |  |  |  |  |  |  |  |  |  |  |  |
| neg | **633,2425** | 9,55 | 60,24 | 4,00 | 1.05E-06 | **0.94** | 5,00 | **C26H40N4O12S** | ChemSpider ID: 435603 |  |  |  |
|  |  |  |  |  |  |  |  | **C38H36N2O5S** | ChemSpider ID: 2696558 | ChemSpider ID: 2814914 | ChemSpider ID: 3307421 | ChemSpider ID: 3333638 |
|  |  |  |  |  |  |  |  | **C36H41O6PS** | ChemSpider ID: 9257879 | |  |  |
|  |  |  |  |  |  |  |  | **C25H46O14P2** | ChemSpider ID: 9942059 |  |  |  |
|  |  |  |  |  |  |  |  | **C42H35NO3P** | ChemSpider ID: 9073105 |  |  |  |
|  |  |  |  |  |  |  |  |  |  |  |  |  |
| pos | **711,1763** | 9,04 | 24,79 | 4,00 | 6.01E-05 | **0.99** | 5,00 | **C31H35O19** | ChemSpider ID: 8525308 | ChemSpider ID: 8946383 | ChemSpider ID: 9825322 | KNApSAcK ID: C00004425 |
|  |  |  |  |  |  |  |  | **C37H34N4O5S3** | ChemSpider ID: 3649944 | ChemSpider ID: 5113057 | ChemSpider ID: 12848863 | ChemSpider ID: 12848928 |
|  |  |  |  |  |  |  |  | **C44H26N2O8** | ChemSpider ID: 9322048 | |  |  |
|  |  |  |  |  |  |  |  | **C36H30N4O10S** | ChemSpider ID: 8050705 |  |  |  |
|  |  |  |  |  |  |  |  |  |  |  |  |  |
| neg | **839,1729** | 8,54 | 112,44 | 4,00 | 5.29E-08 | **0.93** | 1,00 | **C46H34N2O10S2** | ChemSpider ID: 9073421 |  |  |  |
|  |  |  |  |  |  |  |  |  |  |  |  |  |
| neg | **711,1457** | 5,38 | 9,09 | 4,00 | 0.00332 | **0.72** | X |  |  |  |  |  |
|  |  |  |  |  |  |  |  |  |  |  |  |  |
| neg | **823,1989** | 9,56 | 22,81 | 4,00 | 3.32E-05 | **0.75** | X |  |  |  |  |  |
