# Supplementary material for: Metabolomics Unravel Contrasting Effects of Biodiversity on the Performance of Individual Plant Species
Source: PLoS One. 2010 Sep 7;5(9):e12569. doi: 10.1371/journal.pone.0012569 (PMC2935349; doi:10.1371/journal.pone.0012569)
Supplement: Table S2 — Number of plots per species-richness level where plant material of the five studied species was collected. Plots are classified in community without/with legumes for non-leguminous herb species, and communities with/without non-legumes for legume species. The number of plots in which the original seed mixtures contained the studied species is given in parentheses. (0.03 MB DOC) [file pone.0012569.s002.doc]

Table S2

| Species | Monocultures | 2 species | 4 species | 8 species | 16 species | 60 species |
| --- | --- | --- | --- | --- | --- | --- |
| **Non-leguminous herb species** |  |  |  |  |  |  |
| *Bellis perennis* L. | 1/0 (1/0) | 1/0 (1/0) | - | 1/0 (1/0) | 1/2 (1/2) | 0/3 (0/4) |
| *Leontodon autumnalis* L. | 1/0 (1/0) | - | 0/1 (0/1) | 1/1 (1/1) | 0/1 (1/2) | 0/0 (0/4) |
| *Knautia arvensis* (L.) J.M. Coult. | 1/0 (1/0) | - | 1/2 (1/2) | 1/2 (1/2) | 1/1 (1/1) | 0/4 (0/4) |
| **Legume species** |  |  |  |  |  |  |
| *Lotus corniculatus* L. | 0/1 (0/1) | 0/1 (0/1) | 0/1 (0/1) | 4/0 (4/0) | 4/0 (4/0) | 4/0 (4/0) |
| *Medicago x varia* Martyn | 0/1 (0/1) | 1/0 (1/0) | 1/1 (1/1) | 0/1 (0/1) | 3/0 (3/0) | 4/0 (4/0) |
